# Machine Learning-Based Intelligent Scoring System for English Essays under the Background of Modern Information Technology

**DOI:** 10.1155/2022/6912018

**Published:** 2022-05-23

**Authors:** Shaoyun Fu, Hongfu Chen

**Affiliations:** Minjiang University, Fuzhou, Fujian, China

## Abstract

This work is to reduce the workload of teachers in English teaching and improve the writing level of students, so as to provide a way for students to practice English composition scoring independently and satisfy the needs of college teachers and students for intelligent English composition scoring and intelligently generated comments. In this work, it firstly clarifies the teaching requirements of college English classrooms and expounds the principles and advantages of machine learning technology. Secondly, a three-layer neural network model (NNM) is constructed by using the multilayer perceptron (MLP), combined with the latent Dirichlet allocation (LDA) algorithm. Furthermore, three semantic representation vector technologies, including word vector, paragraph vector, and full-text vector feature, are used to represent the full-text vocabulary of English composition. Then, a model based on the K-nearest neighbors (kNN) algorithm is proposed to generate English composition evaluation, and a final score based on the extreme gradient boosting (XGBoost) model is proposed. Finally, a model dataset is constructed using 800 college students' English essays for the CET-4 mock test, and the model is tested. The research results show that the semantic representation vector technology proposed can more effectively extract the lexical semantic features of English compositions. The XGBoost model and the kNN algorithm model are used to score and evaluate English compositions, which improves the accuracy of the scores. This makes the management of the entire scoring model more efficient and more accurate. It means that the model proposed is better than the traditional model in terms of evaluation accuracy. This work provides a new direction for the application of artificial intelligence technology in English teaching under the background of modern information technology.

## 1. Introduction

Nowadays, the Internet industry is developing rapidly, and the continuous improvement of modern information technology drives the progress of human civilization. As an important information carrier, English is the most widely used language in the world. Accordingly, learning English has become a necessary requirement, and English proficiency certificate has become a must for every college student [1].

Modern information technology is a comprehensive computer and telecommunication technology based on modern microelectronic technology, which can collect, process, store, disseminate, and use various sensor signals such as sound, image, text, and numbers [2], and its core is informatics. Modern information technology includes enterprise resource planning (ERP), Global Positioning System (GPS), and radio frequency identification (RFID), so it can learn from RFID knowledge and application of the above aspects. Modern information technology is a technology group with a wide range of contents, including microelectronic technology, optoelectronic technology, communication technology, network technology, sensing technology, control technology, and display technology. Machine learning is a multidisciplinary interdisciplinary major, covering knowledge of probability theory, statistics, approximate theory, and complex algorithms. A technology that uses computers as a tool and is committed to real-time simulation of human learning methods and divides existing content into knowledge structures to effectively improve learning efficiency [3]. Machine learning uses algorithms and models to incrementally improve the ability to accomplish specific tasks. Machine learning builds mathematical models of sample data, called “training data,” to make predictions or decisions without being explicitly programmed to perform a task. Machine learning algorithms can be applied in fields such as e-mail filtering, network intruder detection, and computer vision [4, 5].

The existing research mainly focuses on using the keywords in the composition to evaluate whether the composition is off-topic, extracting the excellent vocabulary in the composition as the basis for scoring the composition and scoring the composition by checking the grammar, which lacks the evaluation of the composition. The innovation of this work lies in the vector representation of the composition vocabulary, sentences, and the full text, so as to obtain the description vector for the full text of the composition and score and evaluate the article.

In this work, an intelligent scoring system for English composition is constructed using machine learning network technology. It discusses the grading requirements of college English composition, uses machine learning technology to construct a three-layer neural network model (NNM) model, and applies three semantic representation vector technologies to represent English composition words, paragraphs, and full-text vectors. The evaluation and scoring model of English composition is proposed, and the proposed model is trained and tested using the English composition data set of students' mock test. The innovation of this work is to use the framework of evaluating English papers to combine three text representation technologies such as word2vec, pos2vec, and paragraph2vec to extract multidimensional semantic features, which provides a reference for generating essay evaluation and scoring. The similarity model incorporating XGBoost (extreme gradient boosting), TF-IDF (term frequency—inverse document frequency), TR (TextRank), and kNN (K-nearest Neighbors) algorithms is used for scoring to increase the accuracy and efficiency of scoring. This research provides a vision to promote more efficient teaching.

## 2. Construction of the Scoring System for English Essays

### 2.1. English Teaching in Colleges and Universities

English is a basic compulsory course for college students in China. It mainly teaches English language and application skills and includes multimedia teaching and listening and speaking teaching. Currently, there are three levels of minimum requirement, general requirement, and high requirement on teaching, and the minimum requirement needs 340 hours. Generally, the 340 hours are distributed in four semesters, namely, 90 hours per semester and 5 hours per week on average, including 3 hours for comprehensive understanding and 2 hours for listening and speaking. Nowadays, colleges and universities do not put much attention on English writing, which needs some changes [6].

### 2.2. Machine Learning

Machine learning focuses on how computers simulate the behavior of humankind and animals. From a statistical standpoint, it establishes a model based on historical data and then uses the model to predict the distribution of data. This requires that test data and training data must be identically distributed. Its basic feature is that it tries to mimic the patterns in which neurons in the brain transmit and process information. Representatively, it is used in computer vision and natural language processing [7]. Obviously, deep learning is strongly related to neural network in machine learning, or we can call deep learning improved neural network algorithms. The main idea of deep learning is to simulate human neurons, and after each neuron receives information and processes it, it can be passed to all adjacent neurons [8, 9].

Artificial neuron is a mathematical model created by imitating the biological neuron. The artificial neuron receives the given signal of the previous neuron, and each given signal will attach a weight. Under the combined action of all weights, this neuron will show a corresponding activation state [10–12], expressed as follows:

(1) $$\begin{array}{c} f\left(x\right)=\overset{n}{\underset{i=1}{\sum }}x_{i}w_{i}, \end{array}$$

where *f*(*x*) is the final output, *x*_*i*_ is the input signal, and *w*_*i*_ represents the weight corresponding to the input signal, with *n* groups in total.

When a neuron receives the input signal, it will give an output. Each neuron has a corresponding threshold. If the sum of the input is greater than the threshold, it enters the activated state. Otherwise, it is in the inhibited state. Artificial neuron has several transfer functions expressed as follows [13, 14]:

(a) Linear function:
  (2) $$\begin{array}{c} f\left(x\right)=kx. \end{array}$$
(b) Slope function:
  (3) $$\begin{array}{c} f\left(x\right)=\alpha \left(x\geq \theta \right),\\ f\left(x\right)=kx\left(-\theta <x<\theta \right),\\ f\left(x\right)=-\alpha \left(x\leq \theta \right). \end{array}$$
(c) Transition function:
  (4) $$\begin{array}{c} f\left(x\right)=\alpha \left(x\geq \theta \right),\\ f\left(x\right)=\beta \left(x\leq \theta \right). \end{array}$$

The transfer function should be selected according to the specific application range. Linear function can amplify the output signal, nonlinear slope function can prevent the impact of degraded network performance, and S-type function is used for the hidden layer.

#### 2.2.1. MLP (Multilayer Perceptron) Model

**Figure 1:**
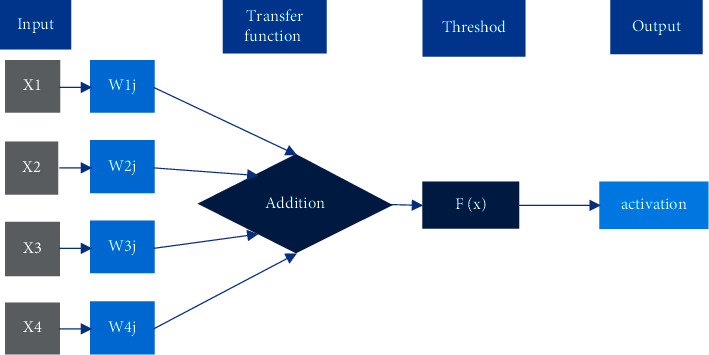
The schematic diagram of the MLP neural network.

The multilayer perceptron model is to obtain the vector of the modeling target, and the basic structure of the multilayer perceptron MLP can be obtained based on the biological neuron model. However, the learning algorithm of the perceptron cannot be directly applied to the parameter learning of the multilayer perceptron model. superior. Therefore, the original proposed learning scheme is described as follows. Except for the last neuron, the weights of all other neurons are fixed in advance, and the learning process is just to use the perceptron learning algorithm to learn the weight coefficients of the last neuron. In fact, this is equivalent to transforming the original feature space into a new feature space through the first layer of neurons. Each neuron in the first layer constitutes one dimension of the new space, and then the perceptron learning algorithm is adopted to construct a linear classifier in the new feature space. A neural network training network takes a feature vector as input and passes this vector to the hidden layer. The result is then calculated through the weights and excitation functions and passed to the next layer until it is finally passed to the output layer. The weights, synapses, and neurons of each layer are computed and learned by training the ANN algorithm. Layers of MLP are fully connected, that is, any neuron in the upper layer is connected with all neurons in the next layer [15, 16], and Figure 1 shows its structure.

It is noted that the neural network has three basic elements: weight, bias, and weight of activation function. The strength of connections between neurons is represented by weights, the size of which represents the bias of possibility. Bias is set to correctly classify samples and is an important parameter in the model, that is, to ensure that the output value cannot be activated randomly. Activation function: it functions as a nonlinear mapping, which can limit the output of neurons within a certain range, generally between (−1, 1) and (0, 1). The most commonly used activation function is the sigmoid function, which can map the number of (−∞, +∞) to the range of (0, 1) [17].

#### 2.2.2. Three-Layer NNM (Neural Network Model)

**Figure 2:**
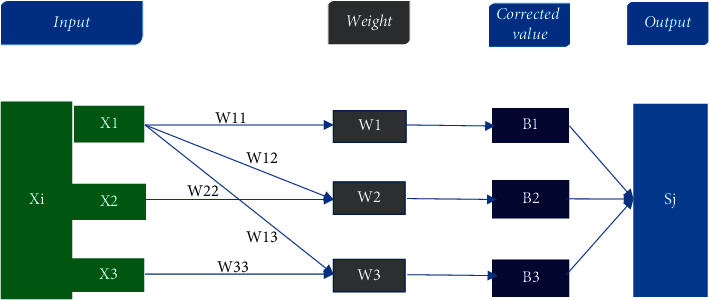
The schematic diagram of the three-layer NNM.

A three-layer NNM is composed of an input layer, a hidden layer, and an output layer, which are interconnected by modifiable weights. The neural network constructed on this basis is composed of three input layers, three hidden layers, and one output layer. The hidden layer unit takes a weighted sum of its inputs to form a net activation. In other words, net activation is the inner product of the input signal and weight of the hidden layer [18, 19], as shown in Figure 2. Each neuron in the neural network can be regarded as a logistic regression model. A three-layer neural network is a composite of a three-layer logistic regression model, except that there is only one neuron in logistic regression. Generally, the input layer and the hidden layer have multiple neurons, and the output layer corresponds to a logistic regression unit or a softmax unit, or a linear regression model. Figure 2 is the schematic diagram of the three-layer NNM:

#### 2.2.3. LDA (Latent Dirichlet Allocation) Model

LDA topic model is a document generation model, which is an unsupervised machine learning technique. It thinks that a document has multiple topics, and each topic corresponds to different words. The construction process of a document is to first select a topic with a certain probability and then select a certain word under this topic with a certain probability, thus generating the first word of the document. Repeating this process continuously will generate the entire article (of course, it is assumed that there is no order between words, that is, all words are stacked in a large bag in disorder, called a bag of words. This method can make the algorithm relatively simplified.). The use of LDA is the inverse process of the above document generation process that is to find out the topics of this document and the words corresponding to these topics according to an obtained document.

LDA believes that the distribution of topics and words conforms to a polynomial distribution because the probability of different words appearing in a topic is different. If there is only one word, it complies with binomial distribution, but it cannot be only one word, so it naturally conforms to the polynomial distribution. The research gives probability distributions for words according to topics, that is, to give the possibility of words belonging to a certain topic. It does not depend on the sequential relationship between words, and the polynomial distribution has been a good description of this relationship. LDA believes that the distribution between articles and topics also conforms to a polynomial distribution because the probability of an article belonging to a certain topic needs to be analyzed based on the occurrence possibility of words [20]. In this work, the MLP technology is combined with the LDA algorithm to construct NNM as an untrained model.

### 2.3. Representation of the Word Vector

**Figure 3:**
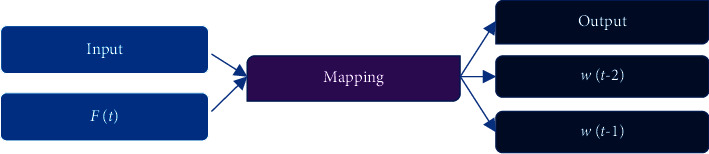
Schematic diagram of the principle of the word2vec model.

Word vector is the basic structure of text. A good word vector can make semantically similar words gather together, which facilitates the subsequent text classification, text clustering, and other operations. Here, the word2vec model is used. The core of the model is the three-layer NNM, which predicts the words that will appear in the context according to the current words.  Figure 3 is the schematic diagram of the word2vec model [21].

*w*_*i*_ in the figure represents the sequence of given training words. According to the principle of the Skip-gram model, the objective function is established, where *c* represents the number of words in the surrounding context of *w*_*i*_ and *c* is proportional to the scope of context. A larger scope means a greater *c* and more time the model needs to run. *p* represents the language probability set and is usually expressed by the Softmax function. The sentences with length L are expressed according to word frequency. *p* is expressed as follows and used to search high-frequency words quickly, thus reducing the difficulty of calculation.

(5) 1T∑t=1T∑−c≤j≤c,j≠0lgpwi+jwi,pw/wi=expvw′vTw1∑w=1Wexpvw′vTw1,=∏j=1Lw−1σnw,j+1=chnw,jvnw,j′vTwi,σx=11+exp−x,

where *v*_*w*_ and *v*_*w*_′, respectively, represent the input and output vectors of the word *w*, *W* represents the total words of the context within the search scope, and *n*(*w*, *j*) represents the path from *j* to the root node and it is calculated as follows.

(6) $$\begin{array}{c} v_{w}^{\prime }=b+Wv_{w}+U\;\tanh\left(d+Hv_{w}\right),n\left(w,j\right)=\text{root},n\left(w,L\left(w\right)\right)=w, \end{array}$$

where tanh is the activation function of the neuron, and *b*, *d*, *W*, *U*,  and *H* are the parameters to be solved. SGD (stochastic gradient descent) [22] and GA (genetic algorithm) [23] are used to seek optimal solutions. The part of speech vector will convert the word sequence *wi* into part of speech sequence *psi*. Word feature vector mainly investigates the rationality of word collocation.

SGD is a simple but very effective method, which is mostly used for the learning of linear classifiers under convex loss functions such as support vector machines (SVM) and logistic regression. SGD has been successfully applied to large-scale and sparse machine learning problems often encountered in text classification and natural language processing. GA originates from computer simulation studies of biological systems. It is a random global search and optimization method developed by imitating the biological evolution mechanism in nature, drawing on Darwin's theory of evolution and Mendel's theory of genetics. Its essence is an efficient and parallel global search method, which can automatically acquire and accumulate knowledge about the search space during the search process and adaptively control the search process to obtain the best solution.

### 2.4. Representation of the Paragraph Vector

**Figure 4:**
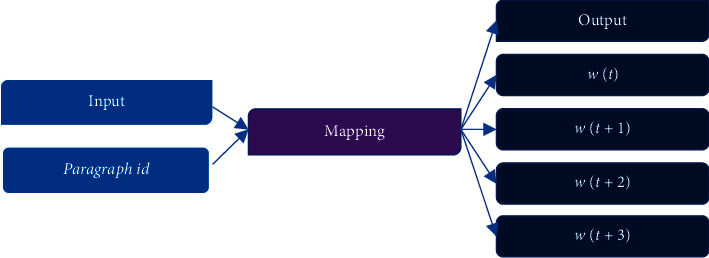
The schematic diagram of the network model.

Paragraph representation conforms to the calculation principle of the paragragh2vec, and MLP model is used to obtain the vector of modeling target. Paragraph ID is added to paragraph2vec, making all sentences or paragraphs have their unique identity.  Figure 4 shows the network model principle [24].

### 2.5. Representation of the Full Text

**Figure 5:**
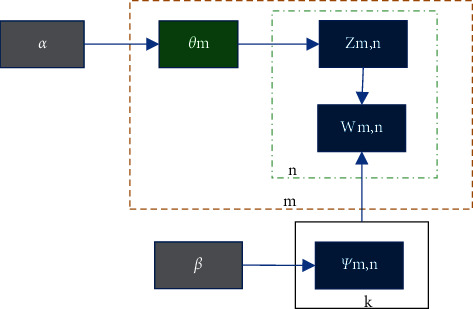
The calculation process of LDA parameters.

LDA is a generative Bayesian probability model, including three parts for words, topics, and documents.  Figure 5 shows the calculation process of its parameters.

The parameters in Figure 5 follow the relationship expressed as follows, where *p* is a function, and *w*, *α*, *β*, and *θ* are all parameters to be solved [25].

(7) $$\begin{array}{c} p\left(w|\alpha ,\beta \right)=\left(\int p\left(w_{n}\theta ,\beta \right)|\right)d\theta ,\\ =\int p\left(\theta |\alpha \right)\left(\prod_{n=1}^{N}\underset{z_{n}}{\sum }p\left(z_{n},\beta \right)p\left(w_{n}|z_{n},\beta \right)\right)d\theta \\ =\frac{\Gamma \left(\sum_{i}\alpha_{i}\right)}{\prod_{i}\Gamma \left(\alpha_{i}\right)}\int \prod_{i=1}^{k}\theta_{i}^{aj-1}\left({\prod_{n=1}^{N}\overset{k}{\underset{i=1}{\sum }}\overset{V}{\underset{j=1}{∐}}\left(\theta ,\beta_{ij}\right)}^{w_{n}}\right)d\theta \;,\\ p\left(\theta |\alpha \right)=\frac{\Gamma \left(\sum_{i}\alpha_{i}\right)}{\prod_{i}\Gamma \left(\alpha_{i}\right)}\theta_{1}^{a_{1}-1}\ldots \theta_{k}^{a_{k}-1}. \end{array}$$

We use the LDA model to represent the full text.  Table 1 shows the production process of an article and the calculation steps of LDA model [26].

### 2.6. XGBoost Algorithm Model

XGBoost is an optimized distributed gradient boosting library designed to be efficient, flexible, and portable. XGBoost provides parallel tree boosting to solve many data science issues quickly and accurately. In terms of large-scale data in the industry, the distributed version of XGBoost has extensive portability and supports running in various distributed environments, making it a good solution to the problem of large-scale data in the industry. Equations (8) and (9) are the objective functions of each parameter.

The objective functions of parameters are expressed as follows:

(8) $$\begin{array}{c} y_{i}^{\prime }=\overset{K}{\underset{k=1}{\sum }}f_{k}\left(x_{i}\right),f_{k}\in F,\\ L={\underset{i}{\sum }l\left(y_{i}-y_{i}^{\prime }\right)}^{2}+\underset{k}{\sum }\left(\gamma T+\frac{1}{2}\lambda w^{2}\right), \end{array}$$

*f*_*k*_(*x*_*i*_) contains two items: to calculate the influence of the number of nodes *T* of the parameter *γ* on the error and to calculate the influence of node weight *w* of the parameter *λ* on error. The regularization method is used to avoid overfitting due to too many nodes. *y*_*i*_′ represents the output value of the composition model, *y*_*i*_ represents the standard value of the composition model, *f*_*k*_(*x*_*i*_) is the tree function, *x*_*i*_ is the input value of the function, and *L* is the objective function of each parameter [27].

### 2.7. kNN Algorithm Model

The kNN classification algorithm is one of the simplest algorithms in data mining classification technology, and its guiding idea is to infer its category from its neighbors. The realization principle of the kNN classification algorithm is explained as follows. In order to judge the category of the unknown samples, the distances between the unknown samples and all the known samples are calculated with the samples of all known categories as the reference, and the K known samples with the closest distance to the unknown samples are selected. According to the voting rule that the minority obeys the majority, the unknown samples and the kNN samples are classified into one category. The kNN classification algorithm only determines the category of the sample to be classified according to the category of the nearest one or several samples, rather than the method of discriminating the category domain to determine the category to which it belongs, so the kNN method is more suitable than other methods for the sample set to be divided with more overlapping or overlapping class domains.

TF-IDF (term frequency—inverse document frequency) is a statistical method used to evaluate the importance of words in articles or in context.  Table 2 shows the general idea to score essays [28].

The importance of words is represented by the number of digits but is the opposite of the frequency in the corpus. Search engines often use TF-IDF weight to measure the level of relevance between files and user questions. In addition to TF-IDF, search engines also use ranking based on link analysis to determine the order in which files appear in search results.

The TR (TextRank) algorithm is a graph-based algorithm to extract keywords and rank abstracts. It is improved from Google's PR (PageRank) algorithm. It can extract keywords using the co-occurrence information (semantic) between words. It can extract key words and key phrases from a given text and extract key sentences from the text.

### 2.8. Test Results of the Scoring System

800 essays were randomly selected from the homework of students in colleges and universities, with a vocabulary of 160–210 words. Two teachers scored each essay separately to obtain the average score. Then, the number of essays in each score segment was counted. 800 essays were divided into 5 groups (160 for each), of which 4 groups were randomly selected as the training set and the remaining 1 group was used as the test set. Five rounds of cross-validation were performed on training and testing, and the evaluation index was recorded each time. Finally, the average of these scores was taken as the final score.

The intelligent scoring model constructed in this work is compared with the traditional gradient boosting decision tree (GBDT) method and the effect of the SVM method. The GBDT method trains the base classifiers in a serial manner, and there are dependencies between the base classifiers. Its basic idea is to stack the base classifiers layer by layer, and it will give higher weights to the samples misclassified by the previous layer of the base classifier when each layer is training. During testing, the final result is obtained according to the weighting of the results of each layer of classifiers. The principle of GBDT is very simple. The sum of the results of all weak classifiers is equal to the predicted value, and then the next weak classifier is to fit the residual of the error function to the predicted value (this residual is the error between the predicted value and the true value). SVM is a binary classification model that maps the feature vector of an instance to some points in the space. The purpose of the SVM is to draw a line that “best” distinguishes the two types of points, so that if new points come later, and the line can also make a good classification. SVM is suitable for small and medium data samples, nonlinear, high-dimensional classification. The classical SVM algorithm only provides two-class classification algorithms, but it is generally necessary to solve multiclass classification in the practical application of data mining. It can be solved by a combination of multiple second-class support vector machines. There are mainly one-to-many combination mode, one-to-one combination mode, and SVM decision tree; then, it is solved by constructing a combination of multiple classifiers. The main principle is to overcome the inherent shortcomings of SVM and combine the advantages of other algorithms to solve the classification accuracy of multiclass problems.

The precision, recall, and F1-score are compared. The F1-score is the harmonic mean of precision and recall, and its calculation formula is

(9) $$\begin{array}{c} F_{1}=\frac{2*\left(\text{precision}*\text{recall}\right)}{\left(\text{precision}+\text{recall}\right)}. \end{array}$$

Mean square error (MSE) is a method to measure the difference between two estimators, as follows. *E* is the expectation, and *D* is the variance.

(10) $$\begin{array}{c} \text{MSE}\left(\theta^{\prime }\right)=E{\left(\theta^{\prime }-\theta \right)}^{2},\\ \text{MSE}\left(\theta^{\prime }\right)=E{\left\{\left[\left(\theta^{\prime }-E\left(\theta \right)\right)\right]+\left[E\left(\theta^{\prime }\right)-\theta \right]\right\}}^{2},\\ \text{MSE}\left(\theta^{\prime }\right)=D\left(\theta^{\prime }\right)+{\left[E\left(\theta^{\prime }\right)-\theta \right]}^{2}. \end{array}$$

The Pearson correlation coefficient (PCC) is used to represent the linear correlation between two variables: *X* and *Y*. In the following equation, *cov* represents the covariance, *σ* represents the mean, and *μ* represents the standard deviation.

(11) $$\begin{array}{c} \rho_{X,Y}=\frac{\mathrm{cov}\left(X,Y\right)}{\sigma_{X}\sigma_{Y}}=\frac{E\left[\left(X-\mu_{X}\right)\left(Y-\mu_{Y}\right)\right]}{\sigma_{X}\sigma_{Y}}. \end{array}$$

## 3. Intelligent Scoring System for English Essays Testing and Analysis

### 3.1. The Categories and Scores of English Essays in the Experiment

**Figure 6:**
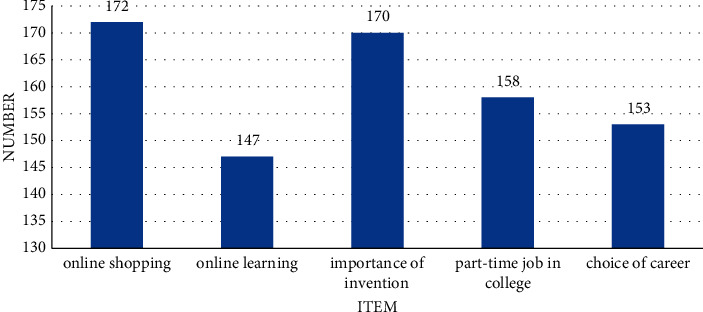
The number of essays belonging to each category.

**Figure 7:**
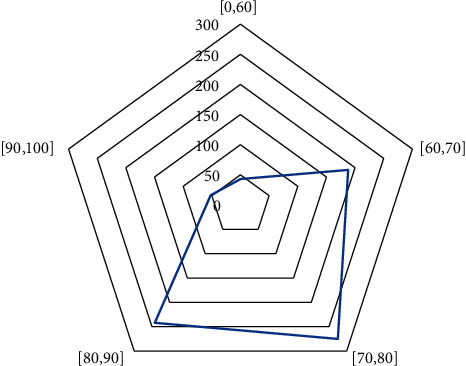
The number of essays in each score range.

Figure 6 shows the number of essays belonging to various categories, and Figure 7 shows the number of essays in each score range.

Figure 6 shows the five types of online shopping, online learning, the importance of investment, university part-time jobs, and career choice. The number in each category is 172, 147, 170, 158, and 153, respectively. Overall, there numbers do not change largely. Figure 7 shows the number of essays in all score ranges. There are 43 essays in [0, 60], 188 in [60, 70], 275 in [70, 80], 242 in [80, 90], and 52 in [90, 100]. It suggests that there is no significant difference in the choice of essay topics by students, and the proportion of students with scores in the [70, 80] and [80, 90] is close to 250, of which the people in the [70, 80] range most, satisfying normal distribution.

### 3.2. Performance of Different Scoring Models

**Figure 8:**
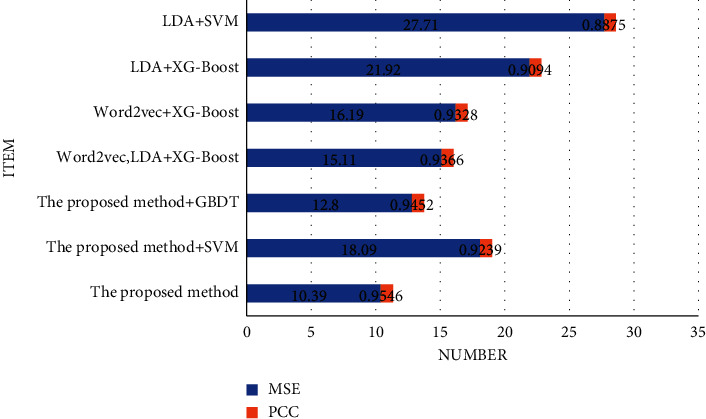
MSE and PCC results of different methods.

Figure 8 shows the MSE (mean square error) and PCC (Pearson correlation coefficient) results of various essay scoring methods.

As shown in Figure 8, the method adopted in this work shows the smallest mean square error, which is only 10.39. After the traditional vector machine SVM method is added to the method proposed, the MSE rises to 18.09; after the GBDT method is added, the MSE rises to 12.80. The smaller the mean square error of the scoring method, the better. The MSE of the traditional scoring method is much higher than that of the scoring method proposed. The proposed method shows the highest PCC, reaching 0.9546; after the method proposed is combined with the traditional method, the PCC decreases. The larger the PCC, the better the performance of the intelligent scoring model, indicating that the method proposed has the highest correlation with the smallest error in the manual teacher scoring results, so the performance of the method is the best.  Table 3 shows the results of precision, recall, and F1-score.

According to Table 3, the proposed method combines the TF-IDF method and TR method to obtain the average score of each essay. It has the best accuracy, recall rate, and F1-score. Its F1-score of 80% is much higher than the other two methods, and its average accuracy is over 80%, while that of the other two methods is less than 70%. Among them, the precision, recall rate, and F1-score of the TR + kNN method are below 70%, while the F1-score value of the TF-IDF + kNN method is 65%, the recall rate is 63%, and the precision is 68%, which are lower than the other two methods. Therefore, the effect of the algorithm model proposed is obviously better than that of the mainstream algorithms.

## 4. Conclusion

In this work, it establishes an intelligent scoring system for English composition under the background of modern information technology. The NNM model is constructed by using MLP technology combined with the LDA algorithm, and three semantic representation vector technologies are used to represent the full-text vocabulary of English composition. Then, the English composition evaluation model based on the kNN algorithm and the final composition scoring model based on XGBoost are proposed. Finally, 800 English compositions are randomly selected from the four-level examination training compositions in colleges and universities, and the established English composition data set is used for training and testing. Finally, the comparative test results between the method used in this work and the traditional method are obtained. The final practical application results reveal that the results of the English composition intelligent scoring system based on machine learning proposed show the smallest mean square error and the highest accuracy and the highest F-score score compared with the artificial teacher's scoring results. Therefore, the model constructed shows the best performance.

Although it has basically achieved the original expected research goals, the research work still has the following shortcomings due to my limited academic quality. Firstly, it only analyzes the composition for the CET-4 test in colleges and universities. In the future, it will collect the composition data of primary and secondary schools and build an intelligent English composition scoring system suitable for various teaching tasks. Secondly, only a few college students' English compositions are collected. In the future, the scale of the data set will be further expanded, data collection will be carried out on the English compositions of college students of different levels, and the number of training sessions will be increased, so as to obtain better training effects.

## Figures and Tables

**Table 1:** The production process of an article and the calculation steps of the LDA model.

Order	Step	Calculation process
1	To determine the distribution of topics and words	LDA is used to calculate the polynomial distribution of feature words and describe the distribution with parameters
2	To determine the distribution of articles and topics	According to PD (Poisson distribution), the scale of feature words is calculated
3	To randomly determine the number *N* of words in the article	LDA is used to calculate the probability vector of topic distribution
4	If the number of currently generated terms is less than N, go to step 5; otherwise, step 6 is performed	From the *m*th essay (*m* = 1, 2,…, M; and *M* is the total number of essays), a feature word w of a topic is extracted. Then, the expectation maximization method is used to estimate the maximum likelihood of parameters, so as to establish the LDA three-layer model.
5	A topic is generated randomly according to article and topic distribution, and then a word is generated randomly based on topic and word distribution. Next, proceed to step 4.	
6	The article generation is finished	

**Table 2:** The general idea to score essays.

Order	Step
1	The TF − IDF method and TextRank method are used to select typical labels to score essays
2	With general feature vector to represent the essay to be scored and the essay in the data set, their feature vectors are compared for cosine similarity. Then, the *k* value of the kNN algorithm model is evaluated, and typical comment tags of essays with high similarity to the essay to be scored are screened to eliminate the duplicates to form the score.
3	To get the score *C*_*i*_ of the *C*_*i*_ essay, it is necessary to use the TF − IDF method to calculate the weight of word segments and arrange them in descending order. Then, the sequence *K*_*i*_^TF−IDF^ of word segments is obtained. *tf*_*i*,*j*_ represents the result of *i*, *j* by TF method, and *idf* is the result by *IDF* method, as follows: *TF* − *IDF*=*tf*_*i*,*j*_ × *idf*_*i*_=, *n*_*i*,*j*_/∑_*k*_*n*_*k*,*j*_ × lg|*D*|/|{*j* : *t*_*i*_ ∈ *d*_*j*_}|.
4	The TextRank method is used to estimate the weight value of each word segment, and the word segment sequence *K*_*i*_^TextRank^ is obtained in descending order. *d* is a given value, expressed as follows. *TR*=(1 − *d*)+*d* × ∑_*V*∈{*Vi*}_*w*_*ji*_/∑_*V*∈*Out*{*Vj*}_*w*_*jk*_.*K*_*i*(*n*)_^TF−IDF^ represents the *n* position in the TF − IDF sequence, *K*_*i*(*n*)_^TextRank^ represents the *n* position in the *TextRank* sequence, and *K* takes the intersection of the two values to obtain the comprehensive scoring sequence of all essays
5	In the scoring process, essay *i* to be scored and that in the data set are expressed as *v*_*i*_^all^ and *v*_*j*_^all^

**Table 3:** The results of precision, recall, and F1-score.

Method	F1-score	Recall	Precision
The proposed method	0.8	0.78	0.82
TR + kNN	0.68	0.66	0.7
TF-IDF + kNN	0.65	0.63	0.68

## Data Availability

The data used to support the findings of this study are included within the article.
